# Indications and Utility of Posterior Tracheopexy in the Pediatric Population: An Overview of Its Expanding Role in Tracheobronchial Disease

**DOI:** 10.3390/children13020199

**Published:** 2026-01-31

**Authors:** Nicholas Jose Iglesias, Ali A. Mokdad, Nelson Vicente Guevara, Andres Mauricio Corona, Eduardo Alfonso Perez, Carlos Theodore Huerta

**Affiliations:** 1 DeWitt Daughtry Family Department of Surgery, Division of Pediatric Surgery, University of Miami Miller School of Medicine, Miami, FL 33136, USA; 2 Division of Pediatric Surgery, Cook Children’s Health Care System, Fort Worth, TX 76104, USA; ali.mokdad@cookchildrens.org

**Keywords:** tracheomalacia, tracheobronchomalacia, posterior tracheopexy, pediatric surgery

## Abstract

**Highlights:**

**What are the main findings?**

- Posterior tracheopexy improves respiratory symptoms, respiratory infection rates, and ventilatory dependence in patients with tracheomalacia.
- Primary posterior tracheopexy during tracheoesophageal fistula/esophageal atresia (TEF/EA) repair may benefit select patients with posterior tracheal intrusion.

**What are the implications of the main findings?**

- Posterior tracheopexy is a valuable surgical technique for the treatment of tracheomalacia (TM) or the reduction in respiratory morbidity following TEF/EA repair in select neonates.
- Patient evaluation and follow-up should be conducted by multidisciplinary teams to optimize long-term outcomes in patients with complex tracheal diseases requiring posterior tracheopexy.

**Abstract:**

**Background:** Tracheobronchial disease, including tracheomalacia (TM) and tracheobronchomalacia (TBM), is a spectrum of congenital and acquired airway disorders characterized by the collapse of the tracheal or mainstem bronchial walls during expiration, particularly when there are increased intrathoracic pressures. Traditional surgical approaches to treat severe medically refractory TM include anterior approaches, such as aortopexy or anterior tracheopexy. Recently, posterior tracheopexy has emerged to address the widened and mobile posterior tracheal membrane which can cause transient airway obstruction. **Method:** The National Institute of Health, National Library of Medicine, PubMed, and MEDLINE databases were queried for manuscripts related to posterior tracheopexy in the pediatric population. Preoperative diagnostics, anesthetic considerations, operative technique, clinical outcomes, and operative complications were analyzed in each manuscript. **Results:** Patients with severe medically refractory cases of TM who are being considered for posterior tracheopexy should undergo thorough preoperative workup by a multidisciplinary team. Cross-sectional, dynamic thoracic imaging and a “quadruple endoscopy”, incorporating laryngoscopy, dynamic bronchoscopy, distal bronchoscopy, and esophagogastroduodenoscopy (EGD) should be obtained as part of a standardized preoperative assessment. Posterior tracheopexy for pre-existing TM significantly improves respiratory symptoms, respiratory infection rates, brief resolved unexplained events, and ventilatory dependence. Recently, posterior tracheopexy during TEF/EA repair has been described and aims to reduce the risk of patients developing TM, the risk of TEF recurrence, and respiratory morbidity following TEF/EA repair. An ongoing randomized controlled trial may help to elucidate the efficacy of primary posterior tracheopexy in select neonates with TEF/EA. **Conclusions:** Posterior tracheopexy is a valuable surgical technique for the treatment of TM or the reduction in respiratory morbidity following TEF/EA repair in select neonates.

## 1. Introduction

Tracheobronchial disease, including tracheomalacia (TM) and tracheobronchomalacia (TBM), is a spectrum of congenital and acquired airway disorders characterized by the collapse of the tracheal or mainstem bronchial walls during expiration, particularly when there are increased intrathoracic pressures. Congenital TM and TBM are characterized by immaturity of the cartilaginous structures of the lower airway, leading to structural weakness and collapse. In contrast, acquired TM and TBM can present due to extrinsic compression by nearby structures or following surgical procedures, such as tracheoesophageal fistula (TEF) and/or esophageal atresia (EA) repair in patients who already had intrinsic malformation of the tracheal walls during embryogenesis [1,2]. Current reports estimate that 1 in 2100 children are affected, predominantly premature neonates and term infants aged 4–8 weeks [1]. This prevalence is higher among children with TEF and/or EA, reaching up to 87%, with approximately 30% of these children developing severe TM requiring invasive procedures or surgical interventions due to respiratory complications [3].

Traditional surgical approaches to treat severe TM include anterior approaches, such as aortopexy or anterior tracheopexy. These procedures involve the surgical fixation of the trachea or aorta to the sternum, placing an anterior radial traction on the airway, and preventing collapse during breathing [4]. Posterior tracheopexy has emerged due to the widened and mobile posterior tracheal membrane which can cause transient airway obstruction [4,5]. Posterior tracheopexy is a procedure involving the surgical fixation of the posterior tracheal membrane to the anterior spinal ligament to alleviate this obstruction. The posterior tracheopexy technique has been popularized in the past two decades as a management option for TM, with high-volume centers around the globe creating multidisciplinary teams to best tailor the treatment of complex airway diseases [6,7,8,9,10,11,12,13,14,15,16].

Among the first data demonstrating the positive efficacy of posterior tracheopexy in the management of severe TBM was a case series of 20 patients who underwent tracheobronchopexy (of which 10 received isolated posterior tracheopexies) from 2012 to 2014 [17]. Evidence has since grown in support of posterior tracheopexy, with current proposals to use the procedure not only as a secondary intervention for refractory TM but also as a primary adjunct during EA/TEF repair, reflected in the ongoing international multicenter randomized controlled PORTRAIT trial [3,18]. Despite increasingly positive evidence and outcomes, few medical centers perform this procedure. This review sought to examine the role of posterior tracheopexy in the management of TM and TBM, covering patient selection criteria, preoperative diagnostic workup, anesthetic and intraoperative considerations, surgical techniques, and clinical outcomes.

## 2. Materials and Methods

The National Institute of Health, National Library of Medicine, PubMed, and MEDLINE databases were queried for manuscripts published from 2000 to 22 September 2025. Relevant manuscripts were identified using keywords and MeSH criteria, including but not limited to “tracheopexy” OR “posterior tracheopexy” AND “pediatric” OR “neonate” OR “infant” OR “child” OR “adolescent”. Manuscripts were considered for full review if they met the following criteria: pediatric population (≤18 years old) and the patient underwent posterior tracheopexy performed by any surgical technique. Manuscripts were excluded from review if they met the following criteria: adult population (>18 years old), only anterior tracheopexy was performed, point of tracheopexy fixation was not defined, aortopexy alone was performed, or the full manuscript in English language was not available (Table 1). Relevant manuscripts were manually added for review if found to be cited by one of the initially identified manuscripts and deemed relevant to this study. Preoperative diagnostics, anesthetic considerations, operative technique, clinical outcomes, and operative complications were analyzed.

## 3. Results/Discussion

### 3.1. Manuscripts Reviewed

**Figure 1:**
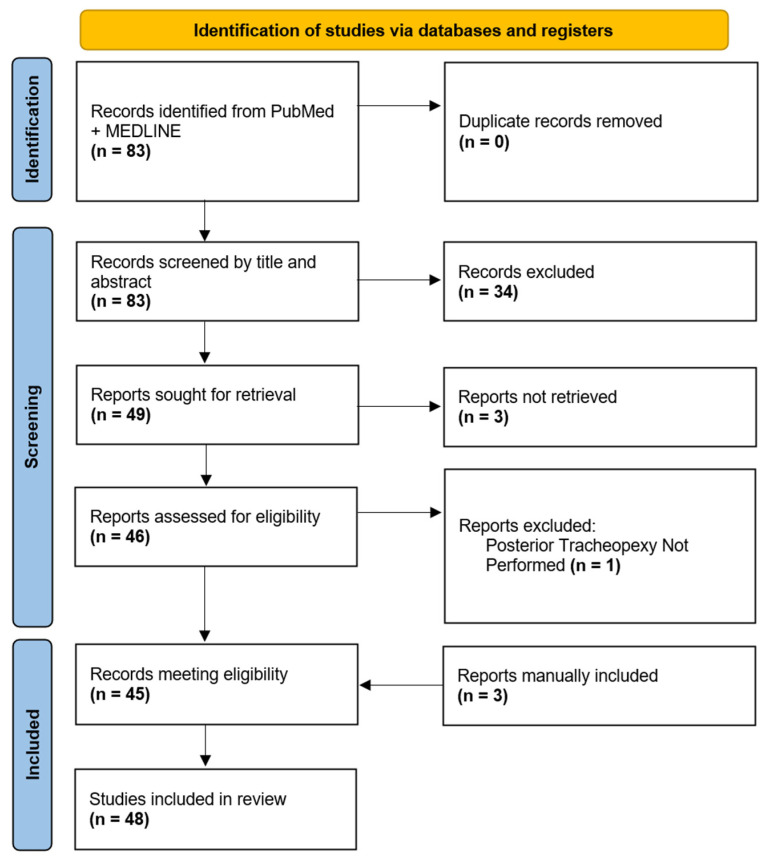
Diagram of Posterior Tracheopexy Manuscripts Selected for Review.

Initial search criteria within PubMed and MEDLINE databases yielded 83 manuscripts for review. After an initial review of manuscript titles and abstracts, 49 manuscripts were selected for a full-text review. Three manuscripts were not available in their English full-text format and one manuscript was excluded due to a lack of posterior tracheopexy. Forty-five (n = 45) of the initially identified manuscripts were eligible for review, and an additional three manuscripts were manually added for review due to a citation in one of the eligible manuscripts and had relevance. In total, 48 manuscripts were ultimately included for the final review (Figure 1).

### 3.2. Patient Selection and Preoperative Diagnostics

#### 3.2.1. Tracheomalacia

Posterior tracheopexy is most commonly performed for the treatment of severe TM that is refractory to maximal medical therapy. While mild to moderate cases of TM are traditionally managed with muco-active nebulizers, inhaled steroids, muscarinic antagonists, antibiotics for respiratory infections, and positive pressure support, many patients suffer from more severe cases [11,19,20,21]. Patients with severe TM/TBM who suffer acute life-threatening events, severe recurrent respiratory tract infections, respiratory obstructions, or prolonged ventilatory dependence should be considered for referral to a tertiary or quaternary center for surgical evaluation [19,21]. Traditionally, medically refractory cases of TM have been managed with tracheostomy and prolonged ventilatory support or aortopexy; however, posterior tracheopexy has recently emerged as a more physiologic surgical intervention for the treatment of TM [22]. Posterior tracheopexy can be a useful adjunct in the treatment of severe TM/TBM when other surgical approaches such as ascending/descending aortopexy alone do not adequately treat TM/TBM [3,4,6,15,23,24,25,26,27,28].

**Figure 2:**
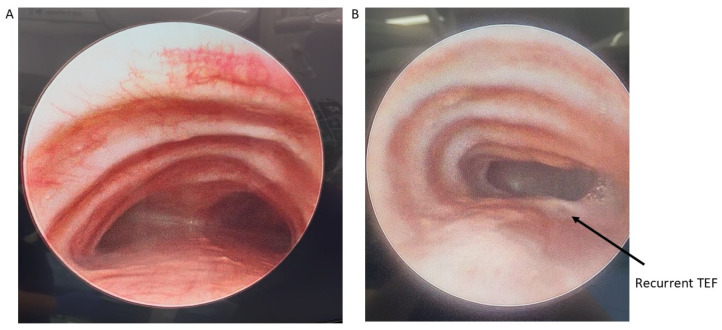
Preoperative bronchoscopy demonstrating posterior tracheal intrusion in patients without (**A**) and with (**B**) recurrent TEF.

Patients with severe medically refractory cases of TM who are being considered for posterior tracheopexy should undergo thorough preoperative workup to ensure proper candidacy for this operation. Beyond the diagnosis and management of underlying associations (e.g., VACTERL in patients who previously underwent TEF/EA repair), patients should undergo “quadruple endoscopy”, incorporating laryngoscopy, dynamic bronchoscopy, distal bronchoscopy, and esophagogastroduodenoscopy (EGD) [9,13,15,19,21,29,30,31,32,33,34,35]. Laryngoscopy facilitates evaluation of the upper airway and any underlying vocal cord dysfunction that may be present preoperatively. Assessment of vocal cord function is particularly important in patients who previously underwent TEF/EA repair as they may have suffered a recurrent laryngeal nerve injury during the prior TEF/EA repair. Dynamic bronchoscopy is the gold standard in diagnosing TM and best informs the obstructive pattern present within the airway (Figure 2). Dynamic bronchoscopy is divided into 3 phases: shallow breathing, cough/Valsalva, and apneic [29]. In the first phase, patients are maintained with deep general anesthesia and allowed to spontaneously ventilate. The spontaneous ventilation phase allows for the assessment of basic airway anatomy, extraluminal compression, malformations, and secretion accumulation [36]. During the second phase, anesthesia is reduced and patients are permitted to cough, Valsalva, or otherwise vigorously breathe [29]. Observation of the airway during these increased intrathoracic pressures reveals the maximum dynamic airway collapse the patient may experience [36]. Dynamic inspection of the tracheal lumen during this period of increased intrathoracic pressure delineates the directionality of tracheal collapse and assists in the selection of an anterior or posterior surgical approach. Observation of an anterior collapse may indicate compression by an aberrant innominate trunk or otherwise aberrant anatomy, whereas posterior intrusion is more indicative of a hypermobile pars membranacea. Finally, for the apneic phase of the dynamic bronchoscopy, patients are deeply sedated again and the airways are distended to 40–60 cm H_2_O, which allows for identification of lesions such as TEF, tracheal diverticulum, and aberrant bronchi [36]. Distal bronchoscopy aids in the diagnosis of more distal TBM that may be less amenable to extraluminal surgical intervention. Standardized endoscopic TM scoring systems have previously been described which quantify the proximal and distal airway mobility seen during a dynamic bronchoscopy [5,6,8,13,15,17,32,33,35,37,38,39]. While these endoscopic TM scoring systems provide a useful framework for characterizing TM disease burden, they should be evaluated in conjunction with other anatomic and imaging findings to direct appropriate surgical management. Finally, EGD allows for the evaluation of possible underlying TEF, anastomotic esophageal stricture, sequela of GERD, and concomitant esophageal lesions. Ideally, all four components of a “quadruple endoscopy” are performed under a single anesthetic event to reduce morbidity for the patient and the logistical burden to families.

**Figure 3:**
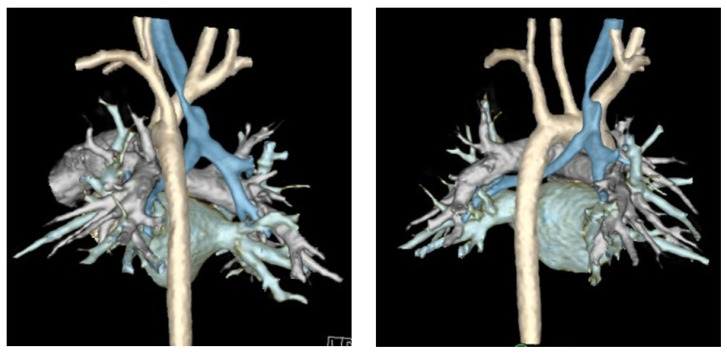
Three-dimensional reconstruction of dynamic airway CT scan. Trachea is highlighted in blue and aorta is highlighted in off-white/cream.

Finally, computed tomography (CT) of the chest with intravenous contrast should be obtained preoperatively to evaluate the great vessel, spinal, tracheal, esophageal, and lung parenchymal anatomy [15,22,29,32,33,38]. Preoperative findings, such as tracheal diverticula, transverse tracheal/bronchial malformations, anterior tracheal compression, or otherwise aberrant anatomy, may redirect surgical plans to involve resection, external splinting, or a combination of these procedures [10]. Cross-sectional imaging methods including Dynamic Volumetric Computed Tomography Angiography (DV-CTA) and contrast-enhanced multidetector computed tomography (MDCT) are preferable for the diagnosis TM/TBM, as they can evaluate the luminal dimensions at both end inspiration and end expiration [5,13,15,19,21,33,34,40,41]. Dynamic airway MDCT and DV-CTA have up to a 91% diagnostic correlation with bronchoscopy [37,41]. Luminal area reduction of >50% during expiration has traditionally often been used as a cutoff for the diagnosis of TM/TBM, and the location of luminal narrowing seen on CT can further be used to guide precise tracheopexy (Figure 3) [19,40,41]. Traditional CT with IV contrast may also be used to measure the lateral and anterior–posterior diameter of the trachea (LAR) at the level of the innominate artery to quantify the severity of tracheal collapse, a metric that has been used to evaluate postoperative outcomes as well [42]. Preoperative cross-sectional imaging also allows for early detection of extrinsic compressions of the trachea by mediastinal masses, descending aorta, vascular rings, or otherwise anomalous cardiovascular anatomy [6,15,38,43]. These should be denoted preoperatively, as this may require involvement of pediatric cardiothoracic surgeons to perform complex vascular reconstructions while the patient is on cardiopulmonary bypass (CPB) [43,44]. Comprehensive workup identifying primarily posterior compression and/or intrusion of the pars membranacea indicates the patient may be a good candidate for posterior tracheopexy [16,20,24,25,45,46].

#### 3.2.2. Primary Posterior Tracheopexy During Esophageal Atresia Repair

Recently, posterior tracheopexy has been proposed as an adjunct procedure to be performed during the index operation for TEF/EA repair. This technique, termed primary posterior tracheopexy, aims to reduce the risk of patients developing TM, the risk of TEF recurrence, and respiratory morbidity following TEF/EA repair, which occurs in up to 89% of children with TEF/EA [3,7,31,47]. Pre- and/or intraoperative bronchoscopy may be used to evaluate posterior tracheal intrusion in patients with TEF/EA. Other centers routinely perform primary posterior tracheopexy in neonates undergoing TEF/EA repair, even in the absence of tracheobronchial collapse on bronchoscopy, in an effort to reduce respiratory morbidity in these patients [2]. Regrettably, there remains significant practice variation with anywhere from 21.5 to 60% of surgeons performing preoperative bronchoscopy for TEF/EA patients. As a result, many candidates may go unrecognized and later develop symptoms of TM/TBM [35]. Proponents of primary posterior tracheopexy cite the more challenging adhered re-operative field present in patients requiring secondary tracheopexy for newly diagnosed TM after TEF/EA repair, which makes primary posterior tracheopexy more optimal [3,7,31]. Similarly to the secondary posterior tracheopexy described above, appropriate patient workup and review by a multidisciplinary team is encouraged. Of note, posterior tracheopexy should not be performed in neonates with TEF/EA if they have midline or anterior descending aorta, as posterior tracheopexy may compress the left mainstem bronchus in patients with these anatomic variants [7]. There is an ongoing international multicenter randomized controlled trial, entitled the PORTRAIT trial, which aims to better elucidate if primary posterior tracheopexy should be incorporated in the standard of care of select TEF/EA patients [3,18].

#### 3.2.3. Recurrent TEF

After TEF/EA repair, up to 15% of patients may subsequently develop recurrent TEF, which often require re-intervention ranging from endoscopic procedures to reoperations [39,48]. In an effort to reduce recurrence rates and the morbidity associated with numerous re-interventions, posterior tracheopexy has been proposed as an adjunct procedure to be performed during TEF/EA repair to reduce TEF recurrence rates [6,7,32,35,39,48]. The purpose of posterior tracheopexy in this context is to physically separate the esophageal anastomosis from the prior fistula site, aiming to reduce friction and the risk of recurrence. The reduction of risks in posterior tracheopexy can be further augmented with the addition of a rotational esophagoplasty, separating suture lines even further [39]. Rotational esophagoplasty alone is also a technique pediatric surgeons can consider, as it has efficacy in reducing TEF recurrence when performed without posterior tracheoplasty [39]. Some even advocate for this combination of procedures to be performed in any child with a recurrent TEF, as it has been found to significantly reduce the risk of TEF recurrence (as little as zero recurrences in 62 patients in one cohort) [39,48].

### 3.3. Anesthetic Considerations

In addition to standard anesthetic considerations for a thoracic procedure, there are several procedural nuances that the anesthesia team should be aware of preoperatively. Intraoperative physiologic considerations include relative hypoventilation, dissection near adjacent nerves, and possible transient aortic compression. Reports from Muñoz et al. and Masaracchia et al. thoroughly discuss anesthetic management during these cases [14,29]. In regard to endotracheal intubation, anesthesiologists should aim to utilize the largest diameter endotracheal tube (ETT) that is safe for placement in the patient. The aforementioned preoperative laryngoscopy may aid in proper sizing of the ETT. Large ETT internal diameters are justified as placement of the posterior tracheopexy sutures requires direct guidance with intraoperative bronchoscopy, which may require up to 1–2 h of continuous partial luminal obstruction [29]. While ETT size is dependent on patient age and size, the use of inner ETT diameters of 3.5–4.5 mm is often described for these cases, and can permit either intermittent or continuous bronchoscopy depending on bronchoscope diameter [7,14,29]. Anesthesiologist should be aware of possible hypercapnia which may occur despite using large ETT diameters during the intraoperative bronchoscopy, causing relative hypoventilation [29]. While most reports of posterior tracheopexy are open approaches, thoracoscopic and robotic approaches are increasingly common for posterior tracheopexy (see Section 3.4). Some centers selectively perform single-lung ventilation in these patients to more readily mobilize the right lung and approach the posterior trachea, especially in children > 1 year old [11,13]. Traditional dual lumen ETTs cannot be used in this capacity as they obstruct the bronchoscopic view required to place the posterior tracheopexy sutures, warranting alternative methods of right bronchial blockade. If pursuing a single-lung ventilation strategy, technologies such as the Arndt endobronchial blocker can be placed extraluminally to maintain unobstructed bronchoscopic visualization of the posterior trachea [11,13,14]. Higher inspired fractions of oxygen (up to 100%) and higher positive end expiratory pressures (PEEP; 5–8 cm H_2_O) may be required to obtain a blood oxygen saturation > 92% during single-lung ventilation [14]. Depending on the physiologic response to the posterior tracheopexy, patients may be considered for extubation in the operating room, with many high-volume centers routinely extubating patients immediately postoperatively [29].

Due to the dissection in the posterior mediastinum and near the aortic arch, surrounding structures, including the recurrent laryngeal nerve, are at risk of intraoperative injury. Numerous centers routinely incorporate recurrent laryngeal nerve (RLN) monitoring, with or without automatic periodic stimulation (APS), during posterior tracheopexy particularly if a neck dissection is contemplated to reduce the risk of injury to this structure [10,29,30,31]. If RLN monitoring is pursued, a neuromuscular blockade should be avoided during dissection or any portions of the cases where the RLN is at risk of injury, as neuromuscular blockade negates the functioning of this system [29,48]. If the surgeon aims to perform a concurrent aortopexy, especially posterior descending aortopexy, careful blood pressure monitoring of the upper and lower extremities is warranted [15,29]. Transient compression of the aorta during surgery may cause a high arm-to-leg blood pressure gradient which should be immediately recognized [15,29]. Finally, as with any other thoracic surgery, regional analgesia should be considered for multimodal pain management, including thoracic epidural, paravertebral block/catheter, or erector spinae block/catheter [29].

### 3.4. Operative Approach

After induction of general anesthesia, patients are traditionally positioned in the left lateral decubitus position, partial rightward prone, or fully prone position depending on the approach, such as open versus minimally invasive [14,15,20,29,35,42,47,49]. The entirety of the patient’s right chest and right neck should be sterilized. Other anatomic considerations, such as anterior tracheal compression, concurrent cardiac disease requiring intervention, or severe left mainstem disease, may warrant sternotomy or a rarely left-sided approach, in which case patients should be placed supine or in right lateral decubitus [4,5,17,50,51]. Patients should be adequately positioned and padded to reduce the risk of positional injuries which may occur during this surgery [13]. Traditionally, posterior tracheoplasty is pursued via a right posterolateral thoracotomy, often in the third or fourth interspace [5,6,7,10,11,15,16,19,24,26,29,32,35,39,45,48]. As recently as 2018, thoracoscopic approaches to posterior tracheoplasty were described, with thoracoscopy and robotic approaches increasing in popularity [2,3,6,7,8,11,12,13,14,20,22,28,32,34,42,46,47,48,49,52,53,54]. After gaining intrathoracic access, the lung should be retracted medially. The esophagus may then be circumferentially dissected and gently retracted using a silastic vessel loop or a small drain [20,49]. Structures within the posterior mediastinum, including the thoracic duct, should be carefully identified and mobilized towards the patient’s left [19,51]. While RLN monitoring is not universally implemented, it may be a valuable adjunct to more safely perform this mobilization, particularly if a neck dissection is performed [10,48,51]. Azygous vein ligation may provide improved exposure and portions of the vein may later be used as an autologous pledget [13,49].

**Figure 4:**
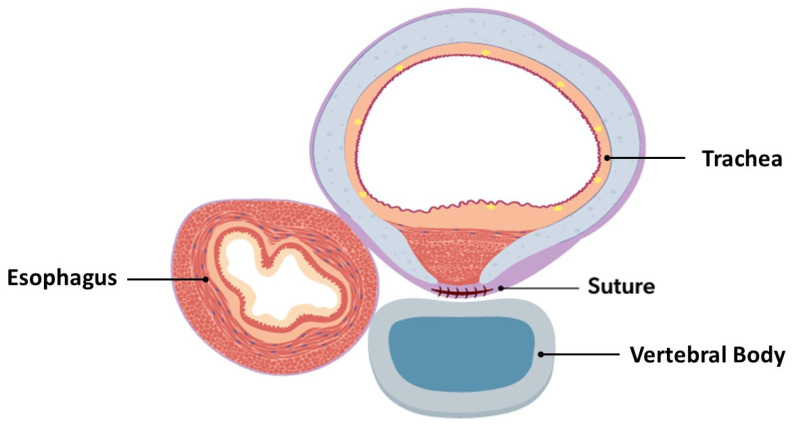
Cross-sectional view of completed posterior tracheopexy with rightward esophageal mobilization.

If not already implemented, intraoperative bronchoscopy, either continuous or intermittent depending on ETT diameter and patient condition, should be initiated [3,5,6,10,13,14,15,16,17,19,20,32,33,35,39,45,46,47,48,49,50,53,54]. Once all structures have been mobilized away from the trachea, the posterior trachea may mobilized and the anterior spinal ligament identified. TEFs or tracheal diverticula that are encountered require transection/resection at this time. The diseased posterior segments of the trachea may then be sutured to the anterior spinal ligament (Figure 4). Direct intraoperative bronchoscopic guidance aids in targeting diseased segments of the trachea, ensuring that the suture is not entering the tracheal lumen, and verifying airway patency improvement after suture placement [3,5,6,10,13,14,15,16,17,19,20,32,33,35,39,45,46,47,48,49,50,53,54]. The number of sutures depends on the patient’s size and severity of tracheal disease; interrupted or horizontal mattress sutures (3-0, 4-0, or 5-0, depending on tracheal dimensions) should be placed approximately every 1 cm, with the use of non-absorbable and absorbable suture materials described [2,4,8,12,22,26,45,46,47,49,50,51]. Sutures may be reinforced with autologous pledgets from a readily available source (pleura, azygous vein, muscular fascia, or pericardium) to better secure the tracheopexy [6,7,10,12,13,15,16,17,26,29,35,38]. Synthetic polytetrafluoroethylene (PTFE) pledgets should be avoided, as they have been found to migrate intraluminally and cause airway compromise [54]. Rightward rotational esophagoplasty may be considered prior to posterior tracheopexy, as this has been found to improve direct apposition of the trachea to the anterior spinal ligament [13,32,38,39,48]. In patients undergoing primary posterior tracheopexy, tracheopexy should precede the esophageal anastomosis [3,8,46]. Upon completion of the posterior tracheopexy, a chest tube or drain placement may be considered due to the risk of postoperative chylothorax [20,49]. Collectively, the complexity of the preoperative evaluation, the numerous intraoperative nuances, and the risk of catastrophic bleeding during dissection near critical structures contribute to a substantial learning curve, underscoring the need for these patients to be managed at centers with appropriate expertise [48].

### 3.5. Outcomes and Complications

**Figure 5:**
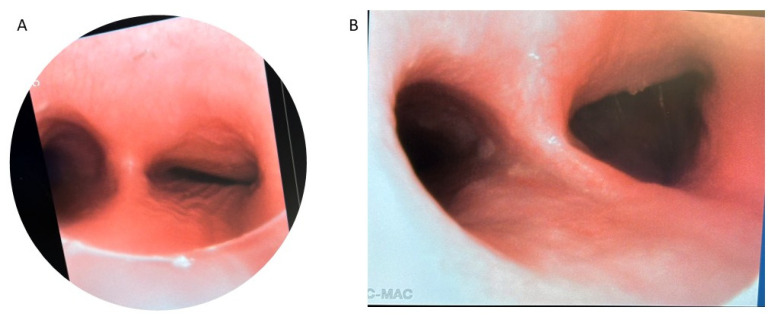
Bronchoscopy of isolated right mainstem bronchomalacia before (**A**) and after (**B**) posterior right mainstem bronchopexy.

After successful posterior tracheopexy, posterior tracheal intrusion is greatly reduced, as most accurately depicted on postoperative bronchoscopy. These findings are also replicated when the technique is applied to more distal airway obstructive diseases, such as isolated right mainstem bronchomalacia (Figure 5). Secondary posterior tracheopexy has been shown to significantly improve respiratory symptoms, respiratory infection rates, brief resolved unexplained events, and ventilatory dependence (Table 2) [5,14,34,35,52]. Up to 90% of patients undergoing posterior tracheopexy experienced significant improvements in respiratory symptoms postoperatively including coughing, noisy breathing, and exercise intolerance [5,13,14,15,29,34,35,45]. The relative risk of respiratory infections was also reduced by >80% in larger case series [5,16,35]. Most patients are completely weaned from mechanical ventilation or noninvasive respiratory support postoperatively due to improved airway patency [14,16,22,35,52]. When performed primarily during TEF/EA repair, posterior tracheopexy has been shown to reduce TEF recurrence risk by up to 100% [39,48]. Furthermore, when performed primarily, patients may have a up to a 68% relative risk reduction in respiratory support dependence at 30 days and a 59% relative risk reduction in respiratory infections [3,7,8,11,42,52]. The operative time for secondary posterior tracheopexy may be as low as 161 min, but there are reports as high as 10.5 h [13,47]. This technique has a learning curve, and the presence of prior TEF/EA and/or esophageal leak with intrathoracic scarring may further complicate and prolong dissection. When performed primarily at the time of TEF/EA repair, the added time to perform primary posterior tracheopexy is reported to be as little as 6 min, which has not been shown statistically to significantly prolong operative times when compared to TEF/EA repair alone [2,7,22,42,46]. Those undergoing secondary posterior tracheopexy often have short ICU courses, relatively short hospital length of stays (often less than one week), and minimal resource utilization [11,49]. Incorporation of a minimally invasive approach can further reduce hospital length of stay to approximately three days in some studies [11]. In patients undergoing primary posterior tracheopexy, there is up to an 11 day reduction in median LOS compared to their counterparts who undergo TEF/EA repair alone, and there is no difference in perioperative complications between cohorts [2,7,42].

**Figure 6:**
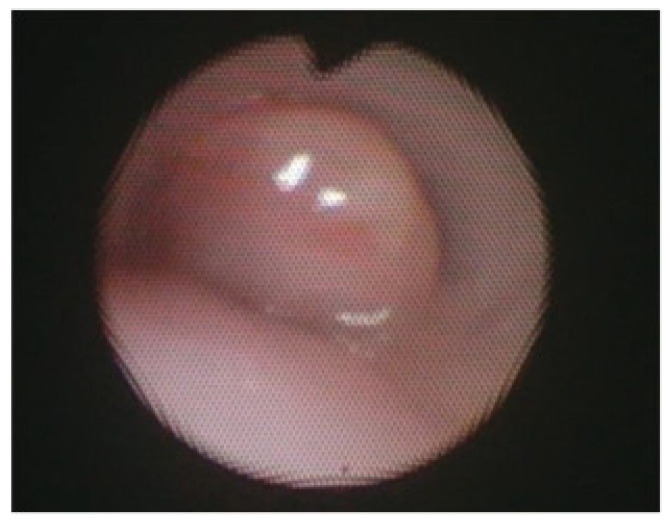
Bronchoscopy showing a granuloma obstruction the tracheal lumen. Reprinted from [3]. Figure is licensed under Creative Commons-BY License.

Postoperative morbidity from posterior tracheopexy largely relates to either the dissection necessary to access the posterior trachea and anterior spinal ligament or changes in esophageal anatomy. Transient or permanent RLN injury can occur during dissection, even despite monitoring, resulting in vocal cord dysfunction. RLN injuries in this setting are most commonly unilateral, they may be managed conservatively and do not require intervention such as tracheostomy; despite this, bilateral injury is a risk, especially in patients with prior mediastinal surgical interventions, that warrants tracheostomy for airway protection [6,26,32,34,35,48]. Surgeons should be aware of preoperative vocal cord dysfunction that may be present in these patients, especially those who previously underwent TEF/EA repair, as a contralateral RLN injury can compromise airway patency [5,35,51]. The risk of RLN injury in posterior tracheopexy underscores the importance of (a) adequate preoperative laryngoscopy, (b) RLN monitoring intraoperatively, and (c) careful dissection to avoid injury. As aforementioned in Section 3.4, mobilization of the thoracic duct is required for adequate visualization and posterior tracheopexy. Chylothorax is a rare but known complication of posterior tracheopexy. Most chylothoraxes which occur after posterior tracheopexy are low volume and can be managed conservatively with dietary modification and drains, which can be placed intraoperatively to monitor for the development of this complication [6,11,20,32,46,49]. Careful suture placement and autologous pledget material selection are also required to reduce the risk of intraluminal granulomas/granulation tissue (Figure 6) [3,8,54]. Intraluminal passage of suture material or the use of synthetic polytetrafluoroethylene pledgets have been linked to the development of these granulomas/granulation tissue and may require re-intervention to relieve respiratory symptoms [3,8,54]. Finally, the repositioned esophagus may cause esophageal dysmotility, as well as narrowing or strictures in up to 46% of patients which may require dilation [13,32,39,48].

Posterior tracheopexy is a complex surgery near numerous vital structures, which places patients at risk of perioperative morbidity and mortality in cases of operative misadventure. These operations are primarily performed at high-volume centers, many of which have comprehensive aerodigestive teams with adequate expertise and resources capable of treating the disease and the perioperative complications [7,8,9,10,11,12,13,14,15,16]. Furthermore, adequate postoperative follow-up is required to evaluate the patient for refractory disease that may require reintervention, including redo posterior airway work, new anterior airway work, or tracheostomy [6]. While the findings of this review highlight the numerous benefits of posterior tracheopexy, it is important to recognize that some lesions may not be adequately treated with posterior tracheopexy such as distal bronchomalacia. Additionally, in a small case series, ¾ patients with bronchopulmonary dysplasia who underwent posterior tracheopexy ultimately required tracheostomy and continued ventilatory support; while there was bronchoscopic improvement in these cases, the location of disease limited clinical improvement to a degree less significant than patients with tracheomalacia after TEF/EA [53].

The presented review has several limitations. As aforementioned, posterior tracheopexy is largely performed at high-volume centers due to the complex preoperative workup, multidisciplinary team management, and numerous intraoperative nuances. Therefore, many studies included in this review are from these high-volume centers and the generalizability of these results may be limited. Furthermore, patient candidacy for posterior tracheopexy is dependent on the center and multidisciplinary team involved in preoperative workup. Further research is required to more clearly direct guidelines for candidacy, such as the degree of posterior tracheal intrusion and other anatomic considerations. While an ongoing randomized controlled trial may help clarify the role of primary posterior tracheopexy in select neonates with TEF/EA, similar efforts are required for secondary posterior tracheopexy.

## 4. Conclusions

Posterior tracheopexy is a valuable surgical technique for the treatment of tracheomalacia or the reduction in respiratory morbidity following TEF/EA repair. Patient evaluation and follow-up should be conducted by multidisciplinary teams to optimize long term outcomes in patients with complex tracheal diseases requiring posterior tracheopexy. Secondary posterior tracheopexy has been shown to improve respiratory symptoms, respiratory infection rates, and ventilatory dependence. Preliminary results suggest primary posterior tracheopexy benefits select TEF/EA patients with posterior tracheal intrusion; however, an ongoing randomized controlled trial may help to elucidate the efficacy of primary posterior tracheopexy in select neonates with TEF/EA.

## Figures and Tables

**Table 1:** Review Inclusion and Exclusion Criteria.

Inclusion Criteria	Exclusion Criteria
Patient Population ≤ 18 Years Old	Patient Population > 18 Years Old
Posterior Tracheopexy to Anterior Spinal ligament	Anterior Tracheopexy
	Point of Tracheopexy Fixation Not Defined
	Aortopexy Alone
	Full Manuscript Not Available in English

**Table 2:** Outcomes after Secondary Posterior Tracheopexy.

Outcome	Frequency	Mean Follow-Up	References
Improvement in Respiratory Symptoms	60–100% of patients (study size between 8 and 100 patients)	3–27 months	[5,11,13,20,32,34,35]
Improvement in Airway Patency on Bronchoscopy/CT	92–100% of patients (study size between 1 and 100 patients)	6–10 months	[5,13,20,35,49,51,52]
Reduction in Recurrent Respiratory Infections	64–85% of patients (study size between 8 and 100 patients)	3 *–5 * months	[5,11,35]
Improvement in Respiratory Support/Oxygen Requirements	25–100% of patients (study size between 1 and 100 patients, worst outcomes with cases of BPD)	5 *–27 months	[5,34,35,51,53]
Length of Stay	Median between 3 and 20.5 days (study size between 8 and 100 patients)	3 *–10 months	[5,11,13,20,35,49]
Recurrent Laryngeal Nerve Injury/Vocal Cord Paresis	1–6% (study size between 25 and 118 patients)	3 *–30 * months	[6,34,35,48]
Chylothorax	2–25% (study size between 8 and 118 patients)	3 *–27 months	[6,11,20]
Granuloma/Granulation Tissue	n = 1	7 months	[54]

* = median follow-up reported.

## Data Availability

No new data were created or analyzed in this study. Data sharing is not applicable to this article.

## Abbreviations

The following abbreviations are used in this manuscript:

TM	Tracheomalacia
TBM	Tracheobronchomalacia
TEF	Tracheoesophageal Fistula
EA	Esophageal Atresia
DV-CTA	Dynamic Volumetric Computed Tomography Angiography
MDCT	Multidetector Computed Tomography
CPB	Cardiopulmonary Bypass
ETT	Endotracheal Tube
PEEP	Positive End Expiratory Pressure
RLN	Recurrent Laryngeal Nerve
